# Traditional Chinese Medicines in Treatment of Patients with Type 2 Diabetes Mellitus

**DOI:** 10.1155/2011/726723

**Published:** 2011-03-17

**Authors:** Weidong Xie, Yunan Zhao, Yaou Zhang

**Affiliations:** ^1^Life Science Division, Graduate School at Shenzhen, Tsinghua University, Shenzhen 518055, China; ^2^Laboratory of Pathological Sciences, Basic Medical College, Nanjing University of Traditional Chinese Medicine, Nanjing 210029, China

## Abstract

Type 2 diabetes mellitus (T2DM) occurs in 95% of the diabetic populations. Management of T2DM is a challenge. Traditional Chinese medicines (TCM) are usually served as adjuvants used to improve diabetic syndromes in combination of routine antidiabetic drugs. For single-herb prescriptions, Ginseng, Bitter melon, Golden Thread, Fenugreek, Garlic, and Cinnamon might have antidiabetic effects in T2DM patients. Among 30 antidiabetic formulas approved by the State Food and Drugs Administrator of China, top 10 of the most frequently prescribed herbs are Membranous Milkvetch Root, Rehmannia Root, Mongolian Snakegourd Root, Ginseng, Chinese Magnoliavine Fruit, Kudzuvine Root, Dwarf Lilyturf Tuber, Common Anemarrhena Rhizome, Barbary Wolfberry Fruit, and India Bread, which mainly guided by the theory of TCM. Their action mechanisms are related to improve insulin sensitivity, stimulate insulin secretion, protect pancreatic islets, and even inhibit intake of intestinal carbohydrates. However, it is very difficult to determine antihyperglycemic components of TCM. Nevertheless, TCM are becoming popular complementary and alternative medicine in treatment of syndromes of T2DM. In the future, it requires further validation of phytochemical, pharmacological, and clinical natures of TCM in T2DM in the future studies, especially for those herbs with a high prescription frequency.

## 1. Introduction

Diabetes mellitus is associated with the increase in cardiovascular diseases and other complications [1–4]. There is increasing incidence of the disease. Type 2 diabetes mellitus (T2DM) occurs in 95% of the diabetic populations. Management of T2DM is a challenge. Plants play an important role in introducing new medicines with antidiabetic activities, such as antidiabetic drugs, biguanidines derives from guanidine of French lilac (*Galega officinalis*). Traditional and modern Chinese medicines are becoming the important sources for the development of antidiabetic drugs [5–10]. There is an increasing need for those T2DM patients intolerant of adverse effects of chemical drugs and/or those who cannot afford expensive medical expenditures in developing countries. 

There are some original or review papers on herbal medicines in treatment of T2DM [5–7]. They supply some useful information of sources, active principles, phytochemistry, and pharmacology of traditional Chinese medicines (TCM) with antidiabetic activities. However, there is still insufficient evidence to draw definitive conclusions about the efficacy of TCM for diabetes. Li et al. systemically list many TCM with antidiabetic effects but have not showed those antidiabetic formula products which are mainstay of TCM [6]. Liu et al. indicate some TCM including single herb prescriptions and formula in treatment of T2DM but most of these herb medicines do not belong to regular products of TCM approved to be used clinically [7]. Though Jia et al. have reviewed the antidiabetic herbal drugs officially approved in China [5] but show only eight antidiabetic herbal formulas, it is not enough to display special aspects of TCM. 

In this paper, we updated and supplemented some reputed TCM approved by the State Food and Drugs Administrator (SFDA) in China mainland in the treatment of T2DM and tried to supply some worthy herb sources and conduct a discussion on how to develop TCM in treatment of T2DM.

## 2. Methods

We searched for papers published in MEDLINE, CNKI, EMBASE, Wiley InterScience, Elselvier databases without language limit by retrieving key words “herb/plant, clinic/clinical, human/patients, type 2 diabetes mellitus” to identify herb medicines in treatment of type 2 diabetes; only those paper with clinical trials will be selected. For Chinese herb formulas, only those listed in the database of products with antidiabetic effect in the official website of http://www.sfda.gov.cn/ will be retrieved. These searches were conducted by two independent examiners. The last date of the search was December 1, 2010.

## 3. Results

### 3.1. Single-Herb Prescriptions

There were only a few reputed single-herb prescriptions found in China. Although those single herb prescriptions had reputed records or/and promising clinical results, they still served as adjuncts or supplements for T2DM patients.

### 3.2. Ginseng

Ginseng together with its roots, stalk, leaves, and berries had significant antihyperglycemic effect in many animal models of T2DM. Some clinical studies indicated that Ginseng is an emerging alternative therapy for T2DM [11–13]. Ginseng significantly decreased insulin resistance and fasting blood glucose (FBG) in T2DM patients [14, 15]. In china, among 30 cases of T2DM treated with Renshen tangtai, an injection contained Ginseng polypeptide and polysaccharides; 86.7% of the patients showed appreciable effect on diabetic symptoms [16]. However, Ginseng had no effect on indices of glucose regulation following acute or chronic ingestion in healthy volunteers [17]. Its active compounds with antihyperglycemic effects included ginsenosides, polypeptide [18], and polysaccharides [19]. Ginseng might exert a antihyperglycemic effect by promoting insulin secretion [11], protecting pancreatic islets [20], stimulating glucose uptake [21], and enhancing insulin sensitivity [15]. Future studies require identifying the component(s) of Ginseng [22]. Ginseng had no significant side effects [15]. However, chronic overdosed administration of Ginseng may suffer from gastrointestinal, mental, cardiovascular, and hormone disorders. Children and pregnant women should be cautious of using this herb.

### 3.3. Golden Thread

Golden Thread is commonly used to treat diabetes in China. Berberine is an isoquinoline alkaloids and the active ingredient of Golden Thread. Berberine had a significant antihyperglycemic effect in both 36 patients newly diagnosed with T2DM and also in 48 poorly controlled patients with T2DM [23]. This effect was comparable to metformin. Berberine increased glucose uptake and stimulates glycolysis by activation of AMP-activated protein kinase (AMPK) [24, 25]. In addition, berberine promoted insulin secretion by modulating glucagon-like peptide-1 release [26]. Berberine promoted beta cell regeneration [27]. In intestinal region, berberine inhibited glucose absorption by suppressing disaccharidase activities [28]. Expect for transient gastrointestinal adverse effects, no significant functional liver or kidney damages were documented.

### 3.4. Bitter Melon

Bitter melon is more commonly used by persons from Asian countries. Bitter melon lowered fasting and postprandial serum glucose levels in T2DM patients [29]. Although bitter melon might have antihyperglycemic effects, data were not sufficient to recommend its use in the absence of careful supervision and monitoring [30]. Major active compounds in this plant contained cucurbitane triterpenoids [31], polypeptide-p, charantin, and vicine [32]. Bitter melon exerted a antihyperglycemic effect by inhibition of protein tyrosine phosphatase 1B (PTP1B), activation of AMPK, increase of glucose transporter type 4 (GLUT4) expression, promotion of the recovery of beta cells [33], and insulin-mimicking action [34]. However, adverse effects of bitter melon included hypoglycemic coma and convulsions in children and headaches [35]. Bitter melon might have additive effects when taken with other glucose-lowering agents. Despite this, no serious adverse effects were reported in all the clinical trials. There were no documentations of death from any cause, morbidity, (health-related) quality of life, and costs [36].

### 3.5. Fenugreek

Fenugreek improved blood glucose control and insulin resistance in diabetic patients [37–39]. The findings of 18 cases of patients showed that FBG, triglycerides, and very low-density lipoprotein cholesterol (VLDL-C) decreased significantly after taking fenugreek seed soaked in hot water [40]. Combined therapy of total saponins of Fenugreek with sulfonylureas hypoglycemic drug lowered the blood glucose level and ameliorated clinical symptoms in 46 cases of T2DM compared with 23 cases of controls [41]. Active components of Fenugreek included trigonelline, nicotinic acid, GII [42], diosgenin [43], 4-hydroxyisoleucine [44], total saponins [45], Fenugreek oil [46], and soluble dietary fibre fraction [47]. Its antihyperglycemic mechanisms were associated with potentiating insulin secretion, increasing insulin sensitivity [48], and inhibiting intestinal carbohydrate digestion and absorption [47]. Fenugreek was relatively safe [45] and also had no genotoxicity [49]. However, we should be cautious of its use combined with aspirin in case of the risk of bleeding [50].

### 3.6. Garlic

Garlic had antihyperglycemic and antihyperlipidemic effects in T2DM patients [51, 52]. In the 4-week double-blinded placebo-controlled study in 60 T2DM patients, Garlic lowered FBG, serum fructosamine, and serum triglyceride levels [53]. Garlic constituents mainly included sulfur-containing compounds (e.g., diallyl trisulfide [54], ajoene [55], S-allyl cysteine sulfoxide [56]) and garlic oil. Garlic improved glycemic control through increased insulin secretion and enhanced insulin sensitivity [54]. Garlic had no significant adverse effects. However, we should be cautious of excess and chronic administration of garlic because of gastrointestinal troubles. If patients suffered from hepatitis, kidney and heart diseases, intake of garlic should be prohibited [57].

### 3.7. Cinnamon

The oral administration of cinnamon per day reduced serum glucose and lipid levels in patients with T2DM, suggesting that the inclusion of cinnamon in the diet of patients with T2DM would reduce risk factors associated with diabetes and cardiovascular diseases [58]. Cinnamon lowered hemoglobin A1c (HbA1C) by 0.83% compared with usual care alone lowering HbA1C by 0.37% in patients with T2DM in a randomized, controlled trial [59]. Its active components contained cinnamaldehyde [60] and naphthalenemethyl ester derivative [61]. Its antihyperglycemic effects were worked by promoting insulin release, enhancing insulin sensitivity, and increasing glucose disposal. Also, Cinnamon seemed to exert insulin-like effects through regulation of PTP1B and insulin receptor kinase [62]. Cinnamon had no significant adverse effects but may be of concern when used in excessive amounts [63].

### 3.8. Traditional Chinese Formulas

In this paper, we listed 30 traditional Chinese herbal formulas approved by China SFDA (Table 1). Although these formulas are listed in China SFDA website, we had no authorized access to their unpublished clinical data. Only part of them is published in the open journals but mostly in Chinese journals. Most of these Chinese formulas were used in combination with routine compounds such as glibenclamide or metformin and indicate that they had better effects in lowering blood glucose and improving diabetic symptoms than routine drugs alone in T2DM patients. In two Chinese formulas, glibenclamide was directly added and considered as a component of formulas. A very small amount of Chinese formulas were used as monotherapy in slight or mild cases of T2DM. 

**Figure 1:**
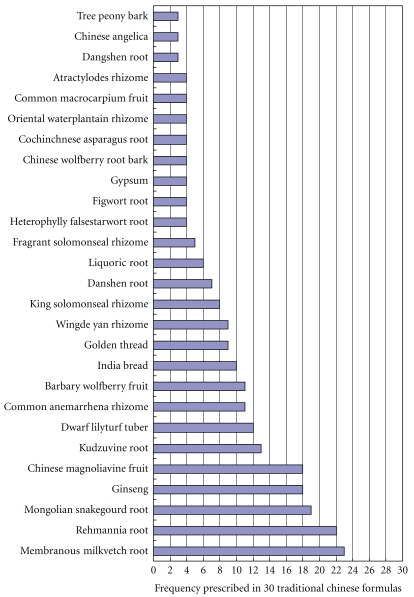
Frequency of herb medicines prescribed in 30 traditional Chinese formulas.

Among 30 formulas above, we listed 3 or more than 3 frequently prescribed herbs in Figure 1. Top 10 of the most frequently prescribed herbs were Membranous Milkvetch Root, Rehmannia Root, Mongolian Snakegourd Root, Ginseng, Chinese Magnoliavine Fruit, Kudzuvine Root, Dwarf Lilyturf Tuber, Common Anemarrhena Rhizome, Barbary Wolfberry Fruit, and India Bread. According to the theory of TCM, these herbs could be grouped as *Qi* (energy-) invigorating, *Yin* (body fluids-) nourishing, heat (body heat-) clearing and stasis-reducing (improving blood circulation and kidney function) drugs (Table 2). For *Qi*-invigorating drugs, the most frequently prescribed herb was Membranous Milkvetch Root (Huang qi, 23 in 30). For *Yin*-nourishing drugs, the most frequently prescribed herb was Mongolian Rehmannia Root (Di huang, 22 in 30). For heat-clearing drugs, the most frequently prescribed herb was Common Anemarrhena Rhizome (Zhi mu, 12 in 30). For stasis-reducing drugs, the most frequently prescribed herb was India Bread (Fu ling, 10 in 30)

### 3.9. Yuquan Wan

Yuquan Wan (YQW) has been long used to treat diabetes in Chinese medicines. Among 18 diabetic patients treated with Yuquan Wan for 1 month [64], 72% cases showed significant or moderate improvement in fasting blood glucose and other diabetic symptoms such as thirst and hunger disappeared. In relation to diabetic complications, Yuquan Wan improved the index of kidney injures of early diabetic nephropathy in diabetic patients, which suggested that it would prolong the development of diabetic nephropathy [65]. YQW improved the insulin resistance [66] in patients with T2DM. YQW reduced the levels of the increased proinflammatory cytokines in patients with T2DM [67]. YQW had a significant effect on the pharmacokinetics of metformin hydrochloride in diabetic rats [68]. This might explain why YQW had a better effect than metformin alone. Taken together, YQW mainly improved diabetic complications and exerted a antihyperglycemic effect mediated likely by enhancing insulin sensitivity. No significant adverse effects were reported of YQW.

### 3.10. Tangmaikang Jiaonang

Tangmaikang Jiaonang (TMK) is used to treat T2DM and its complications. There were many clinical reports of TMK with good effects in treatment of T2DM [69, 70], insulin resistance [71, 72], dyslipidemia [73], diabetic peripheral neuropathy, and blood fluid parameters [69, 74]. These results were drawn only by comparing between before and after combined treatment with routine antidiabetic drugs such as sulfonylureas or biguanides. One clinical report showed that TMK had a better effect in treatment of T2DM patients than Xiaoke Wan [75]. TMK could treat T2DM and could reduce hypoglycemia and the dose of insulin at the base of controlled blood glucose [76]. TMK combined with metformin had better effect than metformin alone in newly diagnosed T2DM patients [77]. TMK enhanced the effect of routine drug on diabetic peripheral neuropathy [78]. TMK combined with routine drugs had more improvement in blood fluid parameters than routine drugs alone [79]. TMK mainly improved diabetic complications and exerted an antihyperglycemic effect mediated by increasing insulin sensitivity but mostly used in combination with the regular antihyperglycemic measurements. There were no significant adverse effects reported in the previous studies.

### 3.11. Xiaoke Wan

Xiaoke Wan (XKW) contains several herb medicines and a western compound, glibenclamide. It is used to treat T2DM. Many clinical reports showed that XKW had similar or better antihyperglycemic effects in diabetic patients compared with glibenclamide [80–84]. In these reports, about 82%  (*n* = 61) [80], 94.1%  (*n* = 86) [81], 92%  (*n* = 100) [82], 93.8 (*n* = 300) [83], and 98%  (*n* = 137) [84] of T2DM patients had significant or moderate improvement in hyperglycemia and other diabetic symptoms, respectively. XKW had more improvement in other diabetic symptoms such as thirsty and hungry or complications such as blood lipid and blood fluid parameters than glibenclamide. Besides of stimulation of insulin secretion mediated by glibenclamide (one of components in XKW), XKW enhanced insulin sensitivity likely mediated by promoting adiponectin secretion in T2DM patients [85]. Although XKW was claimed to be more safe than glibenclamide to a certain extent, herb medicine components cannot completely resist the untoward effect of glibenclamide. To be particular, overuse should be avoided this formula contained glibenclamide which easily cause because severe hypoglycemic response after overuse. Indeed, it had a severe hypoglycemic response in 36 cases of T2DM patients [86].

### 3.12. Jinqi Jiangtang Pian

Jinqi Jiangtang Pian (JQJT) had a moderate antihyperglycemic effect in mild or moderate T2DM patients but had no significant effect in severe T2DM patients when it was used alone [87]. Nevertheless, its use combined with positive drug such as glibenclamide had better effects in T2DM patients after the treatment than glibenclamide alone [88], even when those positive drugs could not work well in those patients [87]. In addition, JQJT combined with positive drugs (such as metformin, Acarbose or glibenclamide) might have more improvement in diabetic dyslipidemia [89] and the early development of diabetic nephropathy [90] than positive drugs alone. The effect of JQJT was confirmed in 30 cases of T2DM recently once more [91]. In addition, effective interventions of JQJT on prediabetes were conducting [92] since prediabetes was a growing health concern where a large percentage of these patients develop full T2DM. Its antihyperglycemic action was related to improvement of insulin sensitivity by comprehensive mechanisms, for example, reducing serum lipid, regulating immune functions, enhancing antioxidative systems, and improving micro-circulation and beta-cell function [93]. JQJT had no significant adverse effects in T2DM patients.

### 3.13. Jiangtangjia Pian and Kelening Jiaonang

Jiangtangjia Pian (JTJ) is used to treat T2DM patients. Among 48 cases of T2DM patients, JTJ improved blood glucose control after the treatment combined with antidiabetic drugs such as sulfonylureas, biguanides, and insulin [94]. Interestingly, in this study, blood glucose was poor controlled in these patients of T2DM by using those antidiabetic drugs before the use of JTJ. Furthermore, its single use also had antihyperglycemic effect in 10 newly diagnosed T2DM patients. Herbs in Kelening Jiaonang (KLL) were similar to Jiangtangjia Pian but might have different oral dosage or prepared process. This formula was used to treat T2DM patients. In clinical report, KLL significantly lowered blood glucose level in T2DM patients (*n* = 30) combined with regular antidiabetic drugs after treatment compared with that before treatment [95]. Glibenclamide in combination with KLL in treatment of T2DM patients (*n* = 33) was more effective and less toxic than its single use [96]. KLL for 1 month of oral administration significantly improved blood glucose levels in 30 cases of T2DM patients who administrated regular antidiabetic drugs but had poor control [97]. Also, 8 weeks of treatment of KLL had a significant improvement in blood glucose in those T2DM patients (*n* = 21) with sulfanylurea failure, which indicated that KLL might improve insulin resistance [98]. Both JTJ and KLL reduced the blood glucose and increased body weight in alloxan-induced diabetic mice [99, 100], suggesting that these drugs might exert an insulin-like effects. No significant adverse effects were reported of these herbs.

### 3.14. Xiaotangling Jiaonang

Xiaotangling Jiaonang (XTL) is another formula contained both herb medicines and glibenclamide. It was reported that XTL had significant antihyperglycemic effect in 30 cases of T2DM after treatment compared with that before treatment [101]. Among 44 cases of T2DM, 88.6% of patients showed significant and moderate improvement after XTL treatment while 75.0% of those patients (*n*  =  32) treated with positive control showed the effect [102]. About 98.67% of T2DM patients (*n*  =  150) treated with XTL in combination with metformin showed significant or moderate improvement in diabetic symptoms and other compications (diabetic dyslipidemia and blood fluid parameters) while only 78% of those patients (*n*  =  50) treated with metformin showed similar effects [103]. In addition, management of XTL effectively improved blood glucose control in 66.7% of diabetic patients (*n*  =  24) with secondary failure of sulfanylurea [104]; XTL treatment also increased insulin sensitivity index in T2DM patients (*n*  =  47) compared with glibenclamide (*n*  =  35), which indicated that treatment of XTL could improve insulin resistance in T2DM patients. Antihyperglycemic mechanisms of XTL are related to improve insulin sensitivity in T2DM patients. We should be cautious of their hypoglycemic events since XTL contained glibenclamide as XKW did.

### 3.15. Shenqi Jiangtang Keli

Shenqi Jiangtang Keli (SQJT) is clinically used in T2DM patients. In a clinical study, 82.85% of T2DM patients (*n*  =  35) show appreciable effects after SQJT treatment [105]. SQJT significantly enhanced the antihyperglycemic effect of metformin in 30 cases of T2DM patients [106]. SQJT alone had significant antidiabetic effect in 235 cases of T2DM patients compared with diet or exercise-controlled controls. But most of patients were diagnosed as slight or mild cases [107]. SQJT mainly improved the diabetic syndromes and even exerted a antihyperglycemic effect in T2DM patients with secondary-failure to sulfonylureas [108]. Antidiabetic mechanisms of SQJT are related to improve insensitivity [109] and restore functions of pancreatic islets in T2DM patients [110]. SQJT had no significant adverse effects.

### 3.16. Other TCM

#### 3.16.1. Yangyin Jiangtang Pian

In a clinical study [111], 95.8% of T2DM patients (*n*  =  120) treated with Yangyin Jiangtang Pian (YYJT) in combination with metformin showed a significant and moderate improvement in diabetic symptoms while this effect was done only in 72.5% of the patients treated with metformin (*n*  =  40). YYJT lowered blood glucose, increased insulin levels, enhanced insulin sensitivity, and improved blood fluid parameters in alloxan-induced diabetic rats [111]. No significant adverse effects were documented of YYJT.

#### 3.16.2. Xiaoke Jiangtang Pian

Xiaoke Jiangtang Pian (XKJT) had significant antihyperglycemic in T2DM patients (70% of 30 cases show appreciable effect) [112]. XKJT also showed a significant glucose-lowering effect in alloxan-reduced mice. In acute and subacute toxicological trials, no significant adverse effects were observed in rats or dogs.

#### 3.16.3. Yijin Jiangtang Jiaonang

Among 42 cases of T2DM patients treated with Yijin Jiangtang Jiaonang (YJJT), about 78.6%, 83.3%, and 45.2% of patients showed appreciable decrease in fasting blood glucose, postprandial blood glucose, and HbA1c levels, respectively [113]. In addition, YJJT had significant decrease in blood triglycerides and total cholesterol levels. YJJT might exert antidiabetic activities through enhancing insulin sensitivity. No hypoglycemic events or any significant adverse effect was recorded.

## 4. Discussion and Perspective

### 4.1. The Theory of TCM Plays a Fundamental Role in Prescribing TCM in Treatment of T2DM Patients

**Figure 2:**
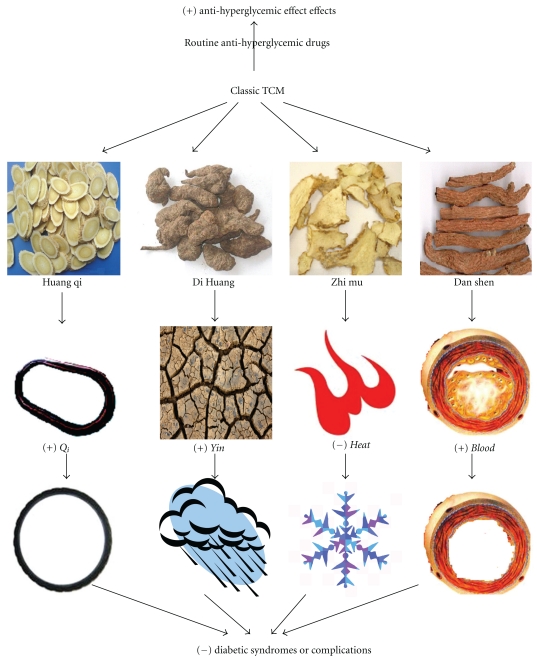
Antidiabetic effects of classic traditional Chinese herbs and their action model in treatment of T2DM patients based on the theory of traditional Chinese medicine.

In this review, for single-herb prescriptions, Ginseng, Bitter melon, Golden Thread, Fenugreek, Garlic, and Cinnamon might have antidiabetic effects in T2DM patients. Among 30 antidiabetic formulas approved by China SFDA, top 10 of the most frequently prescribed herbs are Membranous Milkvetch Root, Rehmannia Root, Mongolian Snakegourd Root, Ginseng, Chinese Magnoliavine Fruit, Kudzuvine Root, Dwarf Lilyturf Tuber, Common Anemarrhena Rhizome, Barbary Wolfberry Fruit, India Bread, which mainly guided by the theory of TCM. In spite of diversity of Chinese formulas, some herb components are frequently presented in formulas. Based on the theory of TCM, most of these Chinese herbs investigated can be grouped as *Qi* (energy)-invigorating, *Yin* (body fluids)-nourishing, *Heat* (body heat)-clearing, and *Stasis* (congested blood circulation or urine)-reducing drugs, and so forth. Diabetes (named Xiaoke in the theory of TCM) is usually associated with the deficiency of both* Qi* (energy) and *Yin* (body fluids) and results in the *Heat* of tissues and blood or urine S*tasis* (congested blood circulation or urine) [114]. This may show the syndromes of Shang Xiao (impairment of the body fluid by the lung-heat), Zhong Xiao (excessive blazing heat in the stomach), and Xia xiao (deficiency of the kidney-*Yin*, deficiency of both *Yin* and *Yang*) in TCM. Those prescribed herbs might be effective to treat the diabetic syndromes in TCM. Although the theory of TCM is difficult to understand, it is very useful to direct us to develop effective traditional Chinese herbs with systemic antidiabetic activities.  Figure 2 indicates the action model of TCM in treatment of T2DM patients. Indeed, the rich and colorful TCM may be prescribed according to the diversity of diabetic syndromes or complications, which is similar to personalized therapy in western medicine [115]. In the future, we might select these highly frequently prescribed herbs as further study in order to disclose their scientific nature.

### 4.2. TCM Serve As Effective Complementary and Alternative Medicine in Treatment of T2DM Patients

As described above, TCM serve as effective complementary and alternative medicine in treatment of T2DM patients although part of results is acquired by poor designs or controls. Most of TCM showed promising results on antidiabetic effects in combination with routine antidiabetic treatment. A very small amount of TCM could alone get satisfied antidiabetic effects in those T2DM patients newly diagnosed or with the failure of routine antidiabetic treatments. In addition, most of TCM are lower cost, more effective for some specific complications, and less adverse effect than regular antihyperglycemic drugs. This may also be the reasons why TCM are popular as complementary and alternative medicine in treatment of syndromes of T2DM. Action mechanisms of TCM mainly involved in improving insulin sensitivity, stimulating insulin secretion, protecting pancreatic islets, inhibiting intake of intestinal carbohydrates, and so on. These actions may play an important role in serving as effective complementary and alternative medicines to routine antidiabetic drugs. 

Taken together, TCM, especially for those herbs with a high prescription frequency, are hopeful to serve as effective complementary and alternative medicine in treatment of T2DM patients based on the theory of TCM. However, active components of TCM are still far from our knowledge since TCM are usually consisted of more than 2 herbs and each herb contains hundreds of compounds. We should establish some appropriate methods to define the active components of TCM and to guarantee stable pharmacological and clinical effects. On the other hand, pharmacological or clinical effects of TCM should be further validated in future studies since part of results is acquired by poor designs or controls.

## Figures and Tables

**Table 1:** Chinese herbal formulas approved by China SFDA.

No.	Products	Ingredients
1	Yuquan Wan	Kudzuvine Root (Ge gen), Mongolian Snakegourd Root (Tian hua fen), Rehmannia Root (Di huang), Dwarf Lilyturf Tuber (Mai dong), Chinese Magnoliavine Fruit (Wu wei zi), Liquoric Root (Gan cao)
2	Tangmaikang Keli	Membranous Milkvetch Root (Huang qi), Rehmannia Root (Di huang), Danshen Root (Dan shen), Medicinal Cyathula Root (Niu xi), Dwarf Lilyturf Tuber (Mai dong), King Solomonseal Rhizome (Huang jing), etc.
3	Xiaoke Wan	Membranous Milkvetch Root (Tian hua fen), Rehmannia Root (Di huang), Mongolian Snakegourd Root (Ge gen), Kudzuvine Root (Huang qi), Wingde Yan Rhizome (Shan yao), Corn Stigma (Yu mi xu), Chinese Magnoliavine Fruit (Wu wei zi), and glibenclamide
4	Jinqi Jiangtang Pian	Golden Thread (Huang lian), Membranous Milkvetch Root (Huang qi), Honeysuckle Flower (Jin ying hua)
5	Jiangtangjia Pian	Membranous Milkvetch Root (Huang qi), King Solomonseal Rhizome (Huang jing), Heterophylly Falsestarwort Root (Tai zi shen), Rehmannia Root (Di huang), Mongolian Snakegourd Root (Tian hua fen)
6	Kelening Jiaonang	Membranous Milkvetch Root (Huang qi), King Solomonseal Rhizome (Huang jing), Heterophylly Falsestarwort Root (Di huang), Rehmannia Root (Tai zi shen), Mongolian Snakegourd Root (Tian hua fen), etc.
7	Xiaotangling Jiaonang	Ginseng (Ren shen), Golden Thread (Huang lian), Mongolian Snakegourd Root (Tian hua fen), Eucommia Bark (Du zhong), Membranous Milkvetch Root (Huang qi), Danshen Root (Dan shen), Barbary Wolfberry Fruit (Gou qi zi), Flastem Milkvetch Seed (Sha yuan zi), White Paeony Root (Bai shao), Common Anemarrhena Rhizome (Zhi mu), Chinese Magnoliavine Fruit (Wu wei zi), glibenclamide
8	Yangyin Jiangtang Pian	Membranous Milkvetch Root (Huang qi), Szechwon Tangshen Root (Dang shen), Kudzuvine Root (Ge gen), Barbary Wolfberry Fruit (Gou qi zi), Figwort Root (Xuan shen), Fragrant Solomonseal Rhizome (Yu zhu), Rehmannia Root (Di huang), Common Anemarrhena Rhizome (Zhi mu), Tree Peony Bark (Mu dan pi), Szechuan Lovage Rhizome (Chuang xin), Giant Knotweed Rhizome (Hu zhang), Chinese Magnoliavine Fruit (Wu wei zi)
9	Shenqi Jiangtang Keli	Ginsenosides from Ginseng stems and leaves, Membranous Milkvetch Root (Huang qi), Rehmannia Root (Di huang), Barbary Wolfberry Fruit (Gou qi zi), Indian Bread (Fu ling), Wingde Yan Rhizome (Shan yao), Mongolian Snakegourd Root (Tian hua fen), Dwarf Lilyturf Tuber (Mai dong), Chinese Magnoliavine Fruit (Wu wei zi), Palmleaf Raspberry Fruit (Fu peng zi), Oriental Waterplantain Rhizome (Ze xie)
10	Jiangtangshu Jiaonang	Ginseng (Ren shen), Barbary Wolfberry Fruit (Gou qi zi), Membranous Milkvetch Root (Huang qi), Manyprickle Acanto-Panax Root (Ci wu jia), King Solomonseal Rhizome (Huang jing), Sharpleaf Galangal Fruit (Yi zhi ren), Oyster Shell (Mu li), Rehmannia Root (Di huang), Prepared Rehmannia Root (Shou Di huang), Kudzuvine Root (Ge gen), Danshen Root (Dan shen), Lychee Seed (Li zhi he), Common Anemarrhena Rhizome (Zhi mu), Gypsum (Shi gao), Gordon Euryale Seed (Qian shi), Wingde Yan Rhizome (Shan yao), Figwort Roo (Xuan shen), Chinese Magnoliavine Fruit (Wu wei zi), Dwarf Lilyturf Tuber (Mai dong), Combined Spicebush Root (Wu yao), Mongolian Snakegourd Root (Tian hua fen), Bitter Orange (Zhi ke)
11	Xiaokeping Pian	Ginseng (Ren shen), Golden Thread (Huang Lian), Mongolian Snakegourd Root (Tian hua fen), Cochinchnese Asparagus Root (Tian dong), Membranous Milkvetch Root (Huang qi), Dan shen root, Barbary Wolfberry Fruit (Gou qi zi), Flastem Milkvetch Seed (Sha yuan zi), Kudzuvine Root (Ge gen), Common Anemarrhena Rhizome (Zhi mu), Chinese Magnoliavine Fruit (Wu wei zi), Chinese Gall (Wu bei zi)
12	Xiaokean Jiaonang	Rehmannia Root (Di huang), Common Anemarrhena Rhizome (Zhi mu), Golden Thread (Huang Lian), Chinese Wolfberry Root Bark (Di gu pi), Barbary Wolfberry Fruit (Gou qi zi), Fragrant Solomonseal Rhizome (Yu zhu), Ginseng (Ren shen), Danshen Root (Dan shen)
13	Qizhi Jiangtang Jiaonang	Membranous Milkvetch Root (Huang qi), Rehmannia Root (Di huang), King Solomonseal Rhizome (Huang jing), Leech (Shui zhi)
14	Xiaoke Jiangtang Pian	Bud of sugarcane node (Zhe ji), King Solomonseal Rhizome (Huang jing), Stevia rebaudiana (Tian ye ju), Mulberry Fruit (Sang shen), Wingde Yan Rhizome (Shan yao), Mongolian Snakegourd Root (Tian hua fen), Ginseng (Ren shen)
15	Zhenqi Jiangtang Jiaonang	Membranous Milkvetch Root (Huang qi), King Solomonseal Rhizome (Huang jing), pearl, Dwarf Lilyturf Tuber (Mai dong), Chinese Angelica (Dan gui), Danshen Root (Dan shen), Chinese Magnoliavine Fruit (Wu wei zi), Heterophylly Falsestarwort Root (Tai zi shen)
16	Yijin Jiangtang Jiaonang	Ginseng (Ren shen), Indian Bread (Fu ling), Large head Atractylodes Rhizome (Bai shu), Cholla Stem (Xian ren zhang), Liquoric Root (Gan cao).
17	Shenhua Xiaoke Cha	Chinese Dodder Seed (Tu si zi), Ginseng (Ren shen), Mongolian Snakegourd Root (Tian hua fen), Common Anemarrhena Rhizome (Zhi mu), Safflower (Hong hua), Gypsum (Shi gao), Green tea, Chinese Wolfberry Root Bark (Di gu pi), Reed Rhizome (Lu gen), Fragrant Solomonseal Rhizome (Yu zhu), Membranous Milkvetch Root (Huang qi), Kudzuvine Root (Ge gen), Platycodon Root (Jie geng)
18	Yusanxiao Jiaonang	Membranous Milkvetch Root (Huang qi), Rehmannia Root (Di huang), Prepared Rehmannia Root (Shou Di huang), Dwarf Lilyturf Tuber (Mai dong), Cochinchnese Asparagus Root (Tian dong), Figwort Root (Xuan shen), Chinese Magnoliavine Fruit (Wu wei zi), Epimedium Herb (Yin yang huo), Danshen Root (Dan shen), Safflower (Hong hua), Chinese Angelica (Dang gui), Golden Thread (Huang Lian), Ginseng (Ren shen), Hairy Antler (Lu rong), Common Anemarrhena Rhizome (Zhi mu), Szechwon Tangshen Root (Dang shen), Mongolian Snakegourd Root (Tian hua fen)
19	Jiangtangning Jiaonang	ginseng (Ren shen), Wingde Yan Rhizome (Shan yao), Gypsum (Shi gao), Common Anemarrhena Rhizome (Zhi mu), Membranous Milkvetch Root (Huang qi), Mongolian Snakegourd Root (Tian hua fen), Indian Bread (Fu ling), Dwarf Lilyturf Tuber (Mai dong), Rehmannia Root (Di huang), Chinese Wolfberry Root Bark (Di gu pi), Corn Stigma (Yu mi xu), Common Macrocarpium Fruit (Shan zhu yu), Liquoric Root (Gan cao)
20	Tangniaole Jiaonang	Mongolian Snakegourd Root (Tian hua fen), Wingde Yan Rhizome (Shan yao), Ginseng (Ren shen), Membranous Milkvetch Root (Huang qi), Rehmannia Root (Di huang), Barbary Wolfberry Fruit (Gou qi zi), Common Anemarrhena Rhizome (Zhi mu), Cochinchnese Asparagus Root (Tian dong), India Bread (Fu ling), Common Macrocarpium Fruit (Shan zhu yu), Chinese Magnoliavine Fruit (Wu wei zi), Kudzuvine Root (Ge gen), Chicken's Gizzard-membrane (Ji nei jin)
21	Yuye Xiaoke Chongji	Membranous Milkvetch Root (Huang qi), Kudzuvine Root (Ge gen), Wingde Yan Rhizome (Shan yao), Common Anemarrhena Rhizome (Zhi mu), Mongolian Snakegourd Root (Tian hua fen), Chicken's Gizzard-membrane (Ji nei jin), Chinese Magnoliavine Fruit (Wu wei zi), Heterophylly Falsestarwort Root (Tai zi shen)
22	Yuquan Pian	Rehmannia Root (Di huang), India Bread (Fu ling), Liquoric Root (Gan cao), Kudzuvine Root (Ge gen), Membranous Milkvetch Root (Huang qi), Dwarf Lilyturf Tuber (Mai dong), Ginseng (Ren Shen), Mongolian Snakegourd Root (Tian hua fen), Smoked Plum (Wu mei), Chinese Magnoliavine Fruit (Wu wei zi)
23	Shenqi Xiaoke Keli	Ginseng (Ren shen), Membranous Milkvetch Root (Huang qi), Wingde Yan Rhizome (Shan yao), Atractylodes Rhizome (Bai shu), Chinese Magnoliavine Fruit (Wu wei zi), Dwarf Lilyturf Tuber (Mai dong), Fragrant Solomonseal Rhizome (Yu zhu), Rehmannia Root (Di huang), Medicinal Cyathula Root (Niu xi), India Bread (Fu ling), Oriental Waterplantain Rhizome (Ze xie), Arctium lappaL (Niu ban zi), bombyx batryticatus (Jiang Can)
24	Ganlou Xiaoke Keli	Prepared Rehmannia Root (Shu Di huang), Rehmannia Root (Di huang), Barbary Wolfberry Fruit (Gou qi zi), Chinese Wolfberry Root Bark (Di gu pi), Common Macrocarpium Fruit (Shan zhu yu), Figwort Root (Xuan shen), Ginseng (Ren shen), Dangshen root (Dang shen), Membranous Milkvetch Root (Huang qi), Chinese Dodder Seed (Tu si zi), Mongolian Snakegourd Root (Tian hua fen), Chinese Angelica (Dang gui), Golden Thread (Huang Lian), Atractylodes Rhizome (Bai shu), Ootheca Mantidis (Sang piao xiao), Cochinchnese Asparagus Root (Tian dong), Dwarf Lilyturf Tuber (Mai dong), Oriental Waterplantain Rhizome (Ze xie), India Bread (Fu ling)
25	Kangji Xiaoke Pian	Ginseng (Ren shen), Golden Thread (Huang Lian), Cortex Phllodedri (Huang bo), Rehmannia Root (Di huang), Prepared Rehmannia Root (Shu Di huang), Barbary Wolfberry Fruit (Gou qi zi), Fragrant Solomonseal Rhizome (Yu zhu), Dwarf Lilyturf Tuber (Mai dong), Chinese Magnoliavine Fruit (Wu wei zi)
26	Xiaokeling Pian	Rehmannia Root (Di huang), Chinese Magnoliavine Fruit (Wu wei zi), Dwarf Lilyturf Tuber (Mai dong), Tree Peony Bark (Mu dan pi), Membranous Milkvetch Root (Huang qi), Golden Thread (Huang Lian), India Bread (Fu ling), Ginseng (Ren shen), Mongolian Snakegourd Root (Tian hua fen), Gypsum (Shi gao), Barbary Wolfberry Fruit (Gou qi zi)
27	Jiantang Jiaonang	Ginseng (Ren shen), Common Anemarrhena Rhizome (Zhi mu), Sankezhen (San ke zhen), Rhizoma Zingiberis (Gan jiang), Chinese Magnoliavine Fruit (Wu wei zi), Ginsenosides from Ginseng stems and leaves
28	Jiangtang Wan	Ginseng (Ren shen), Membranous Milkvetch Root (Huang qi), King Solomonseal Rhizome (Huang jing), India Bread (Fu ling), Atractylodes Rhizome (Bai shu), Golden Thread (Huang Lian), Kudzuvine Root (Ge gen), Chinese Magnoliavine Fruit (Wu wei zi), Chinese rhubarb (Da huang, Liquoric Root (Gan cao)
29	Tangniaoling Pian	Mongolian Snakegourd Root (Tian hua fen), Kudzuvine Root (Ge gen), Rehmannia Root (Di huang), Dwarf Lilyturf Tuber (Mai dong), Chinese Magnoliavine Fruit (Wu wei zi), Liquoric Root (Gan cao), Fried glutinous rice, pumpkin powder
30	Tangle Pian	Mongolian Snakegourd Root (Tian hua fen), Wingde Yan Rhizome (Shan yao), Rehmannia Root (Di huang), Ginseng(Ren shen), Membranous Milkvetch Root (Huang qi), Barbary Wolfberry Fruit (Gou qi zi), India Bread (Fu ling), Common Anemarrhena Rhizome (Zhi mu), Oriental Waterplantain Rhizome (Ze xie), Tree Peony Bark (Mu dan pi), Common Macrocarpium Fruit (Shan zhu yu), wheatgerm

**Table 2:** Classifications of functions of TCM prescribed in 30 traditional Chinese formulas.

Classifications of functions	TCM (Chinese name, times prescribed in 30 formulas)
Invigorating *Qi* (energy)	Membranous Milkvetch Root (Huang qi, 23) Ginseng (Ren shen, 18) Barbary Wolfberry Fruit (Gou qi zi, 11) Wingde Yan Rhizome (Shan yao, 9) Liquoric Root (Gan cao, 6) Heterophylly Falsestarwort Root (Tai zi shen, 4) Atractylodes Rhizome (Bai shu, 4) Dangshen Root (Dang shen, 3)

Nourishing *Yin* (body fluids)	Rehmannia Root (Di huang, 22) Mongolian Snakegourd Root (Tian hua fen, 19) Chinese Magnoliavine Fruit (Wu wei zi, 18) Dwarf Lilyturf Tuber (Mai dong, 13) King Solomonseal Rhizome (Huang jing, 8) Fragrant Solomonseal Rhizome (Yu zhu, 5) Cochinchnese Asparagus Root (Tian dong, 4) Common Macrocarpium Fruit (Shan zhu yu, 4) Cochinchnese Asparagus Root (Tian dong, 4)

Clearing heat	Common Anemarrhena Rhizome (Zhi mu, 12) Kudzuvine Root (Ge gen, 11) Golden Thread (Huang Lian, 9) Figwort Root (Xuan shen, 4) Gypsum (Shi gao, 4) Chinese Wolfberry Root Bark (Di gu pi, 4) Tree Peony Bark (Mu dan pi, 3)

Reducing stasis (improving blood circulation and kidney function)	India Bread (Fu ling, 10) Danshen Root (Dan shen, 7) Oriental Waterplantain Rhizome (Ze Xie, 4) Chinese Angelica (Dang gui, 3)
